# A Polyethylenimine-Linoleic Acid Conjugate for Antisense Oligonucleotide Delivery

**DOI:** 10.1155/2013/710502

**Published:** 2013-06-01

**Authors:** Jing Xie, Lesheng Teng, Zhaogang Yang, Chenguang Zhou, Yang Liu, Bryant C. Yung, Robert J. Lee

**Affiliations:** ^1^Institute of Life Sciences, Jilin University, Changchun, Jilin 130023, China; ^2^Division of Pharmaceutics, College of Pharmacy, The Ohio State University, Columbus, OH 43210, USA

## Abstract

A novel antisense oligonucleotide (ASO) carrier, polyethylenimine conjugated to linoleic acid (PEI-LA), was synthesized and evaluated for delivery of LOR-2501 to tumor cells. LOR-2501 is an ASO targeting ribonucleotide reductase R1 subunit (RRM1). In this study, PEI-LA was synthesized by reacting PEI (Mw ~ 800) with linoleoyl chloride. Gel retardation assay showed complete complexation between PEI-LA and LOR-2501 at N/P ratio above 8. No significant cytotoxicity was observed with these complexes at the tested dosage levels. Interestingly, at N/P ratio of >6, levels of cellular uptake of PEI-LA/LOR-2501 were double that of PEI/LOR-2501 complexes of the same N/P ratio. PEI-LA/LOR-2501 induced downregulation of 64% and 70% of RRM1 at mRNA and protein levels, respectively. The highest transfection activity was shown by PEI-LA/LOR-2501 complexes at N/P ratio of 10. Finally, using pathway specific inhibitors, clathrin-mediated endocytosis was shown to be the principle mechanism of cellular internalization of these complexes. In conclusion, PEI-LA is a promising agent for the delivery of ASOs and warrants further investigation.

## 1. Introduction

Antisense oligonucleotide (ASO) therapy is an emerging therapeutic modality for the treatment of human diseases, including cancer [1–3]. An ASO targets a specific mRNA sequence, reducing its expression [4–6]. A number of ASOs have entered clinical trial [7–9]. However, clinical success for ASO has been very limited, possibly due to the lack of an effective delivery system [10–12]. LOR-2501 is a 20-mer phosphorothioate ASO targeting the R1 subunit of ribonucleotide reductase [13], an enzyme associated with drug resistance. LOR-2501 has shown potent antitumor activities in murine xenograft tumors of the lung, the liver, the ovary, the brain, the breast, and the pancreas. LOR-2501 has been studied in a phase I clinical trial in 2006 for the treatment of prostate cancer [13, 14]. The efficacy of LOR-2501 is dependent on its successful delivery to the cytoplasm. Polyethylenimine (PEI) is a homopolymer with high positive charge density and endosomolytic activity [15–17]. High molecular weight (25 kDa) PEI has frequently been used for gene delivery [18–20]. However, it is fairly cytotoxic [21–23]. Low molecular weight (~800 Da) PEI demonstrates much lower cytotoxicity but is much less active in transfection [22, 24, 25]. Previous studies have shown that conjugating PEI to a lipophilic moiety greatly improved its transfection activity [26, 27]. In the present study, a novel conjugate, PEI-LA was synthesized and evaluated as a carrier for ASO. PEI-LA/LOR-2501 was evaluated in KB cells for biological activity. The mechanism of cellular internalization was also investigated.

## 2. Materials and Methods

### 2.1. Materials

PEI-800 (polyethylenimine 800 Da), triethylamine, and linoleoyl chloride were purchased from Sigma-Aldrich (St. Louis, MO, USA). Sucrose and anhydrous diethylether were purchased from Fisher Scientific (Pittsburgh, PA, USA). CellTiter 96 AQueous One Solution Cell Proliferation Assay System (MTS Assay Kit) was purchased from Promega (Madison, WI, USA). LOR-2501 (5′-CTC TAG CGT CTT AAA GCC GA-3′, fully phosphorothioate substituted) was purchased from Alpha DNA.

### 2.2. Synthesis and Characterization of PEI-LA Conjugate

PEI-LA was synthesized by N-acylation of PEI-800. Briefly, 32 mg PEI-800 was dissolved in 2.5 mL dichloromethane. Triethylamine (50 *μ*L) and then 48 mg linoleoyl chloride, dissolved in 2.5 mL dichloromethane, were slowly added to the stirring solution of PEI. The reaction proceeded for 12 h at room temperature. An excess of diethylether was used to precipitate and then wash the product PEI-LA. Finally, the product was dried under vacuum for 2 h. The product was then analyzed by ^1^H NMR (300 MHz, CDCl_3_). The characteristic proton chemical shifts are in PEI, –NCH_2_–CH_2_N–, **δ** ~ 2.50–3.50 ppm (m, 40H); in LA, **δ** 0.87 ppm (t, 3H, terminal –CH_3_), 1.25 ppm (m, 16H, –(CH_2_)_3_CH_3_ and –(CH_2_)_5_–), 1.75 ppm (b, 2H, –(CH_2_)_5_CH_2_CO–), 2.01 ppm (m, 4H, –CH_2_CHCHCH_2_CHCHCH_2_–), 2.25 ppm (b, 2H, =CHCH_2_CH=), and 5.74 ppm (b, 4H, –CH=CHCH_2_CH=CH–).

### 2.3. Determination of PEI-LA/ASO Complex Formation

An agarose gel retardation assay was conducted to determine the capacity of PEI-LA to form an electrostatic complex with ASO. ASO LOR-2501 was combined with PEI-LA to form complexes at N/P ratios of 1–10. The samples were maintained at room temperature for 30 min and then loaded onto a 1% (w/v) agarose gel containing 0.5 *μ*g/mL ethidium bromide. Electrophoresis was conducted at 100 V for 20 min. The gel was imaged under UV light on an ImageMaster VDS (Pharmacia, Sweden).

### 2.4. Particle Size and Zeta Potential Analysis

Particle size of PEI-LA/ASO complexes was determined by dynamic light scattering (DLS) on an NICOMP submicron particle sizer 370 (Santa Barbara, CA, USA) under the volume-weighted setting. The zeta potential of PEI-LA was determined on a ZetaPALS instrument (Brookhaven Instruments Corp., Worcestershire, NY, USA) after dilution to a volume of 1.4 mL in 0.1X PBS.

### 2.5. Cell Culture

KB cells (a subline of HeLa) were grown in Dulbecco's Modified Eagle Medium (DMEM) (Invitrogen, Grand Island, NY, USA) supplemented with 10% fetal bovine serum (FBS) and 1% penicillin/streptomycin. The cells were maintained at 37°C under a humidified atmosphere containing 5% CO_2_.

### 2.6. Cytotoxicity Assay

KB cells were seeded at a density of 1 × 10^4^ cells/cm^2^ in a 96-well plate 24 h prior to transfection. Cells were washed three times with serum-free media and incubated with PEI-LA/LOR-2501 with varying N/P ratios. Transfection media was removed after 4 h. Fresh culture media was then added, and the cells were incubated at 37°C for an additional 44 h. Then, cell viability was analyzed by MTS assay per the manufacturer's instructions. Briefly, 20 *μ*L MTS solution was added to each well, and the plate was incubated for 1 h at 37°C. Optical density at 490 nm was determined on a standard plate reader. Cell survival was reported as a percentage of the untreated control.

### 2.7. Confocal Microscopy

KB cells were seeded in a chambered cover glass slide overnight and treated with either fluorescent Cy3-LOR-2501 or PEI-LA/Cy3-LOR-2501 for 1 h at 37°C. Cellular nuclei were stained with Hoechst 33342 (Invitrogen, Grand Island, NY, USA) for 5 min at room temperature. Internalization of free or complexed Cy3-LOR-2501 was observed by an Olympus FV1000 confocal microscope (Olympus Optical Co., Tokyo, Japan).

### 2.8. Flow Cytometry

KB cells were seeded in a 24-well plate at a density of 5 × 10^4^ cells/well. After 24 h of incubation at 37°C, cells were washed with serum-free media and treated with PEI-LA/LOR-2501, free LOR-2501, or PEI/LOR-2501 in serum-free media for 1 h. Following treatment, cells were washed with DMEM, harvested, fixed in 4% formalin, and analyzed on an EPIC XL flow cytometer (Beckman Coulter Inc., CA, USA).

### 2.9. Quantitative Analysis of mRNA by RT-PCR

The effect of LOR-2501 complexes on R1 mRNA level was determined by RT-PCR. KB cells were seeded in a 6-well plate at a density of 1 × 10^4^ cells/cm^2^ overnight at 37°C. Cells were treated with PEI-LA/LOR-2501 with varying N/P ratios, free LOR-2501, or PEI/LOR-2501 in serum-free media for 4 h. After treatment, cells were washed three times with 1X PBS, and fresh media was added. Cells were incubated for an additional 44 h. After incubation, media was removed and cells were treated with 1 mL TRIzol reagent per well. RNA was then extracted and purified according to the manufacturer's protocol. RNA was quantified on a NanoDrop 2000 spectrophotometer by measuring OD at 260 nm (Thermo Scientific, Waltham, MA, USA) and converted to cDNA by SuperScript III first-strand synthesis system (Invitrogen, Grand Island, NY, USA) according to the manufacturer's instruction. A PHC-3 thermal cycler (Bio-RAD, Hercules, CA, USA) was used to amplify the cDNA which was then combined with SYBR Green and the appropriate primers for amplification by real-time PCR. R1 mRNA was normalized against β-actin. 

### 2.10. Determination of R1 Protein Expression by Western Blot

Western blot was used to determine the effect of LOR-2501 on R1 protein expression. KB cells, treated as described in the section above, were lysed with a lysis buffer (150 mM NaCl, 50 mM Tris-HCl pH 7.4, 1% v/v NP40) containing a protease inhibitor cocktail (Roche). Protein was quantified by BCA assay (Pierce, Rockford, IL, USA). Then, 20 *μ*g protein samples were loaded onto a 10% SDS-PAGE gel for electrophoresis. Proteins were then transferred to a nitrocellulose membrane. Transferred blots were blocked with 5% nonfat milk in Tris-buffered saline/Tween-20 for 1 h and immunoblotted against the primary antibodies, either goat anti-human R1 (Dako, Carpinteria, CA, USA) or rabbit anti-human GAPDH antibody (Santa Cruz, Santa Cruz, CA, USA), at 4°C overnight. This was followed by incubation with horseradish peroxidase-conjugated rabbit anti-goat IgG (Pierce, Rockford, IL, USA) or goat anti-rabbit IgG (Amersham Biosciences, Piscataway, NJ, USA) for 1 h at room temperature. Blots were developed on an enhanced chemiluminescence (ECL) detection system (GE Healthcare, Waukesha, WI, USA) [28, 29].

### 2.11. Treatment with Endocytosis Inhibitors

KB cells were seeded in a 24-well plate at a density of 5 × 10^4^ cells/well. After 24 h of incubation at 37°C, cells were washed with serum-free media and treated with media containing wortmannin (10 *μ*M), filipin (1 *μ*M), or sucrose (1 M) for 1 h. Wortmannin, filipin, and sucrose are specific inhibitors of macropinocytosis, caveolae/lipid raft-mediated endocytosis, and clathrin-mediated endocytosis respectively. After removing the inhibitor solution, cells were treated with FAM-LOR-2501 or PEI-LA/Cy3-LOR-2501 for 1 h. Cells were then washed twice with PBS and fixed in 4% formalin. Cellular uptake was analyzed on an EPICS XL flow cytometer.

## 3. Results

### 3.1. Characterization of PEI-LA/LOR-2501 Complexes

**Figure 1:**
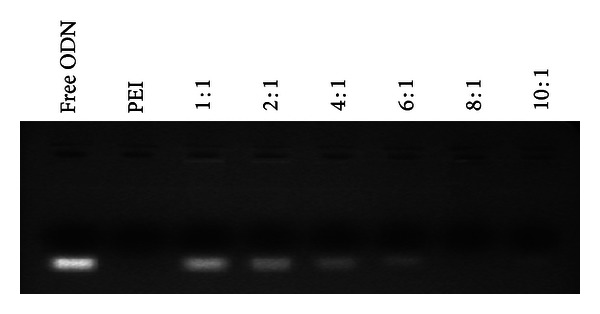
Agarose gel analysis of PEI-LA/LOR-2501 complexes. A series of PEI-LA/LOR-2501 complexes were prepared at varying N/P ratios and analyzed on a 1% agarose gel, as described in Section 2.3.

**Figure 2:**
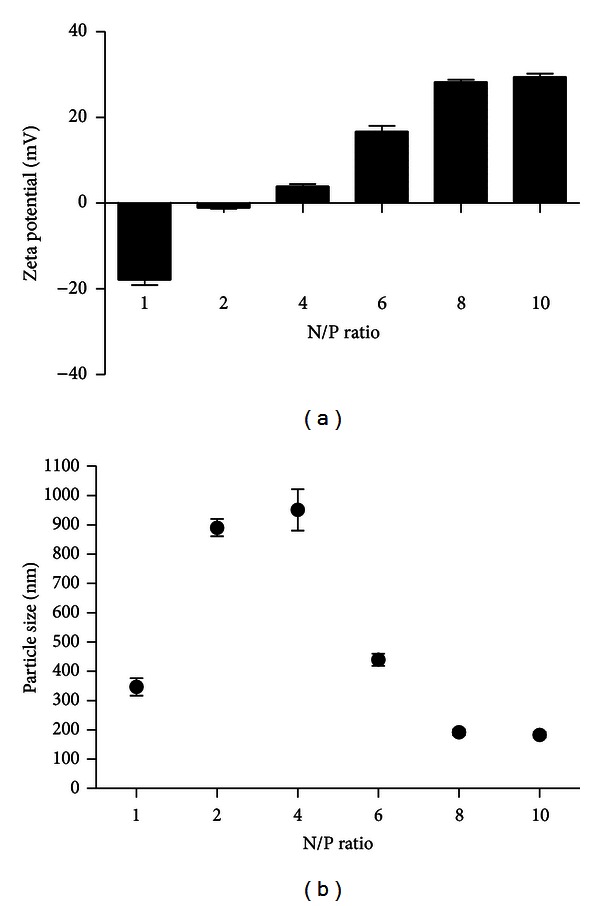
Zeta potential of PEI-LA/LOR-2501 complexes. A series of PEI-LA/LOR-2501 complexes were prepared at varying N/P ratios. Zeta potential and particle size were then measured, as described in Section 2.4.

Degree of complexation between PEI-LA and LOR-2501 was measured by an agarose gel retardation assay. As shown in Figure 1, PEI-LA was able to completely retard LOR-2501 at N/P above 8. The results of particle size and zeta potential analyses are shown in Figure 2. The data showed that increasing the concentration of PEI-LA induced a reversal of zeta potential from negative to positive. Particle size measurement by dynamic light scattering revealed the successful formation of PEI-LA/LOR-2501 particles of under 200 nm in diameter at N/P above 8.

### 3.2. Cytotoxicity of PEI-LA/LOR-2501 Complexes

**Figure 3:**
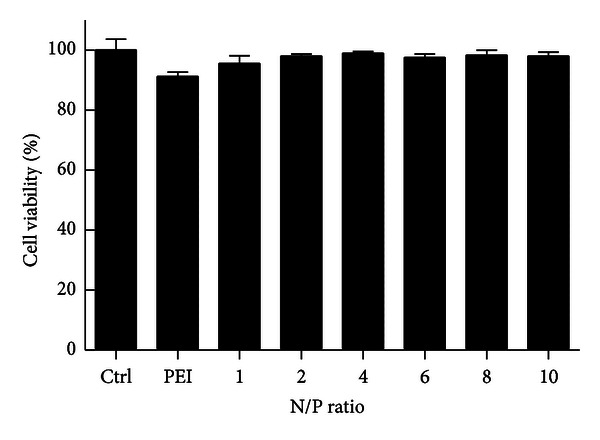
Cytotoxicity of PEI-LA/LOR-2501 complexes. A series of PEI-LA/LOR-2501 complexes were prepared at varying N/P ratios and then added to KB cells. Cell viability was determined by MTS assay at 44 h after transfection.

The cytotoxicity of PEI-LA/LOR-2501 complexes was measured in KB cells by MTS assay. As shown in Figure 3, cell viability was maintained at over 90% across all PEI-LA/LOR-2501 complexes (*P* > 0.05).

### 3.3. Confocal Microscopy

**Figure 4:**
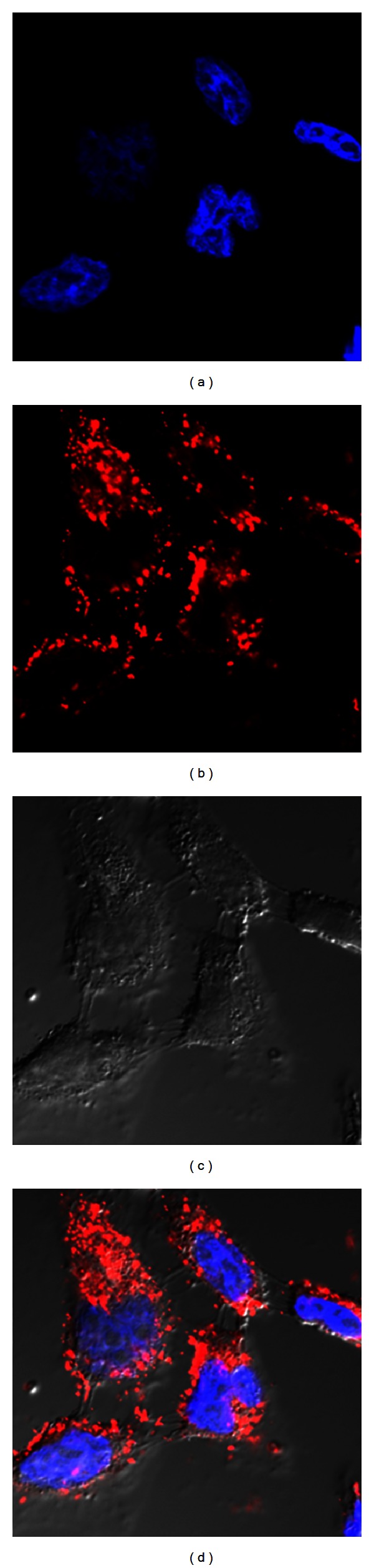
Intracellular localization of PEI-LA/LOR-2501 complexes. KB cells were incubated with PEI-LA complexed to Cy3-labeled LOR-2501 and then evaluated by confocal microscopy. Cy3 fluorescence is shown in red with Hoechst 33342 nuclear stain shown in blue.

In order to investigate the uptake of PEI-LA/LOR-2501 by KB cells, confocal microscopy was employed (Figure 4). The results showed extensive internalization of fluorescently labeled Cy3-LOR-2501 (red, Figure 4(b)) and trafficking to the cytosol. Blue Hoechst 33342 stain was used for observation of the cellular nuclei (Figure 4(a)). A phase contrast image (Figure 4(c)) and an overlay of fluorescent images (Figure 4(d)) are shown as well.

### 3.4. Flow Cytometry

**Figure 5:**
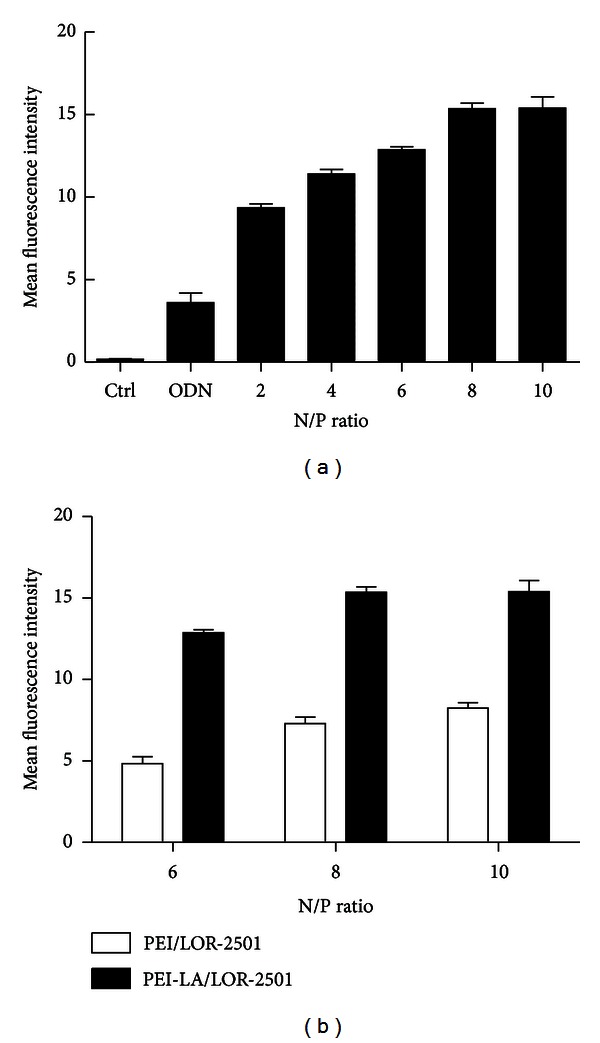
Effect of N/P ratio on cellular uptake of ODN complexes. (a) Cellular uptake of PEI-LA/Cy3-LOR-2501. (b) Cellular uptake of PEI-LA/ASO and of PEI/ASO. A series of PEI or PEI-LA/LOR-2501 complexes were prepared at varying N/P ratios. Cellular uptake was determined by mean fluorescence intensity by flow cytometry.

Similarly, flow cytometry was used to study the uptake of the PEI-LA/LOR-2501 complexes by KB cells. As shown in Figure 5(a), when the N/P ratio of PEI-LA/LOR-2501 complexes was 8, the cells exhibited markedly increased fluorescence intensity relative to those treated with fluorescent-free ASO. Moreover, PEI-LA/LOR-2501 was shown to have higher delivery efficiency than PEI-800 (Figure 5(b)).

### 3.5. Determination of R1 mRNA Expression by Quantitative RT-PCR

**Figure 6:**
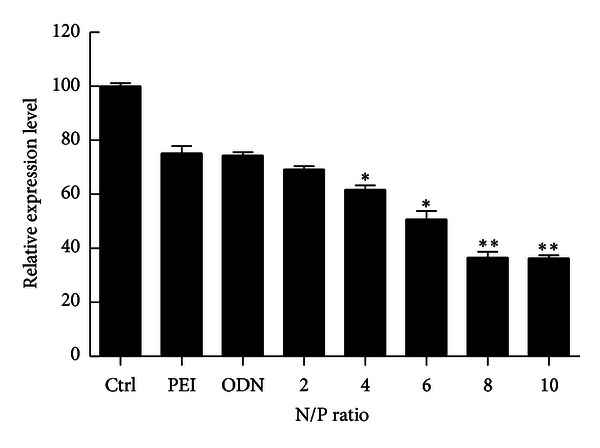
Downregulation of R1 mRNA in KB cells. A series of PEI-LA/LOR-2501 complexes were prepared at varying N/P ratios. R1 mRNA levels were determined by qRT-PCR, relative to β-actin, as described in Section 2.9.

Functional delivery of LOR-2501 with PEI-LA was confirmed by real-time RT-PCR analysis (Figure 6). It was shown that PEI-LA/LOR-2501 induced downregulation of R1 mRNA expression. Greater downregulation was observed at higher N/P ratio. With the control PEI/ASO complexes, mRNA decreased by only 20%. Much greater downregulation of 51% to 64% was encountered for PEI-LA/LOR-2501 complexes at N/P ratios of 6 and 10, respectively. Free LOR-2501 showed only a slight capacity for mRNA downregulation.

### 3.6. Determination of R1 Protein Expression by Western Blot

**Figure 7:**
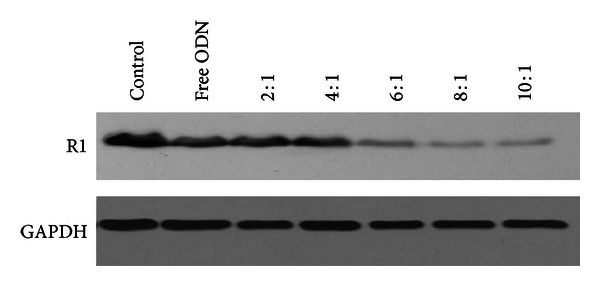
Downregulation of R1 protein in KB cells. A series of PEI-LA/LOR-2501 complexes were prepared at varying N/P ratios. R1 protein and GAPDH levels were determined by Western blot, as described in Section 2.10.

Western blot analysis was conducted to determine the effect of PEI-LA/LOR-2501 on R1 protein level. As shown in Figure 7, free ASO and PEI/LOR-2501 exhibited minimal decreases in R1 protein levels. At higher N/P ratios, the PEI-LA/LOR-2501 complexes showed significant downregulation activity. From N/P 6 to 10, protein downregulation increased from 59% to 70%.

### 3.7. Treatment with Inhibitors

**Figure 8:**
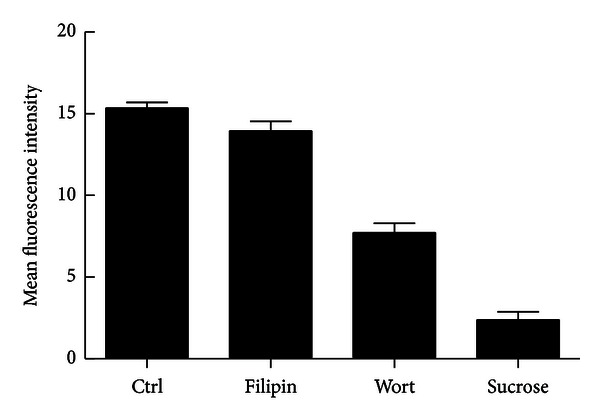
The effect of inhibitor treatment on PEI-LA/Cy3-LOR-2501 uptake. KB cells were treated with various pathway specific endocytosis inhibitors and incubated with PEI-LA/Cy3-LOR-2501 complexes. Cellular uptake was determined by flow cytometry, as described in Section 2.11.

Mechanistic study (Figure 8) of the cellular uptake and trafficking mechanisms is critical to the understanding of the relationship between nanoparticle design and transfection efficiency [30, 31]. Inhibition by filipin, a lipid raft/caveolae-mediated endocytosis inhibitor, reduced uptake by 10%. Inhibition by sucrose, a clathrin-mediated endocytosis inhibitor, reduced uptake by 81%. Inhibition by wortmannin, a macropinocytosis inhibitor, reduced uptake by 52%. Taken together, it is apparent that PEI-LA utilizes all three modes of endocytic transport, with clathrin-mediated endocytosis taking on a leading role in PEI-LA/LOR-2501 delivery. 

## 4. Discussion

Clinical translation of ASO-based therapies has been limited by inefficient delivery of oligonucleotides [32, 33]. In this study, a novel carrier, PEI-LA, was designed and synthesized for use as a delivery agent. PEI-LA has demonstrated excellent properties as a transfection agent. Gel retardation demonstrated total complexation between PEI-LA and LOR-2501 at N/P of 8. Further, no significant cytotoxicity was observed with formulations at the tested dosage levels. 

Surface charge of PEI-LA/ASO is an important factor in determining efficiency of cellular uptake. At the N/P ratio >8 (Figure 2), zeta potentials of PEI-LA/ASO increased and then plateaued at between +25 and +35 mV. PEI-LA/ASO complexes bearing a positive surface potential should facilitate efficient interaction with a negatively charged cell surface [34, 35].

We evaluated the uptake of PEI-LA/ASO in KB cells and found that PEI-LA/ASO generated outstanding transfection activities compared to free ASO. In fact, as we increased PEI-LA/ASO N/P ratio to greater than 6, the cellular uptake of PEI-LA/ASO reached double that of PEI-800 complexes at the same N/P ratio. In addition, PEI-LA/ASO showed much higher transfection efficiency relative to unconjugated low molecular weight PEI as shown by the relative increases in mRNA and protein downreglation. This is consistent with previous reports on hydrophobically modified PEI by Kim et al. and by Teng et al. [26, 27]. The most potent transfection activity was shown with the PEI-LA/LOR-2501 formulation at N/P of 10. The LA moiety has two cis-double bonds, whereas in the previously reported PEI-oleic acid conjugate [27], oleic acid contained a single cis-double bond. The superior transfection activity of PEI-LA is possibly due to the polyunsaturated characteristic of the LA moieties, which may promote bilayer disruption and increased endosomal escape of the ASO following internalization by endocytosis.

The PEI-LA/LOR-2501 complexes showed increased cellular uptake compared to the control PEI/LOR-2501 complexes and greater target downregulatory activities. This may be due to the stabilization of the complexes due to the hydrophobic interactions between the LA moieties. It is interesting that some uptakes were observed with free Cy3-labeled oligos. This may be due to nonspecific cellular uptake due to the use of serum-free media. The observed downregulation was modest. It is worth noting that free LOR-2501 has shown potent antitumor activities in the absence of delivery agent *in vivo* [13], which suggests that it may have some capacity for cellular entry on its own.

Observations by confocal microscopy and data from flow cytometry showed successful delivery of ASO into the cell. Subcellular distribution of the PEI-LA/ASO, as shown in Figure 4, indicated that ASO (red) was localized in the cytoplasm but not in the nucleus (blue). Finally, experiments using internalization inhibitors showed that clathrin-mediated endocytosis was the principle mechanism of entry. Identification of these trafficking mechanisms will be beneficial to further optimization of the formulation.

## 5. Conclusion

PEI-LA is a promising novel agent for the delivery of ASO. The addition of LA to low molecular weight PEI increases delivery efficiency without introducing vehicle-related cytotoxicity. PEI-LA/LOR-2501 was able to efficiently downregulate R1 mRNA and protein levels and, therefore, is a promising candidate for further development. Future studies will evaluate PEI-LA/LOR-2501 in *in vivo* models to evaluate its therapeutic potential.
